# LNMAT1 Promotes Invasion-Metastasis Cascade in Malignant Melanoma by Epigenetically Suppressing CADM1 Expression

**DOI:** 10.3389/fonc.2019.00569

**Published:** 2019-07-03

**Authors:** Kuanhou Mou, Xiang Zhang, Xin Mu, Rui Ge, Dan Han, Yan Zhou, Lijuan Wang

**Affiliations:** ^1^Department of Dermatology, the First Affiliated Hospital of Xi'an Jiaotong University, Xi'an, China; ^2^Department of Clinical Laboratory Medicine, Xijing Hospital, Fourth Military Medical University, Xi'an, China

**Keywords:** LNMAT1, EZH2, CADM1, invasion-metastasis cascade, malignant melanoma

## Abstract

The invasion-metastasis cascade is one of the most important factors relating to poor survival and prognosis of malignant melanoma (MM) patients. Long non-coding RNA lymph node metastasis associated transcript 1 (LNMAT1) is a key regulator in lymph node metastasis of multiple cancer types, but the roles and underlying mechanisms of LNMAT1 in the invasion-metastasis cascade of MM remain unclear. In the present study, we aimed to investigate the expression and function of LNMAT1 in MM. Here, we found that LNMAT1 was upregulated in MM tissues and cells, and its expression levels were further enhanced in MM patients with lymph node metastasis and metastatic MM cells. Using loss-of-function assays, we found that LNMAT1 promoted cell migration and invasion and lung metastasis in MM *in vitro* and *in vivo*. Moreover, we found that cell adhesion molecule 1 (CADM1), the established tumor suppressor in MM, was the downstream target of LNMAT1. Mechanistically, LNMAT1 epigenetically suppressed CADM1 expression by recruiting EZH2, the key regulator of trimethylation of histone H3 at lysine 27 (H3K27me3), to the CADM1 promoter, resulting in transcriptional inhibition of CADM1. Lastly, rescue assays demonstrated that LNMAT1 promoted cell migration and invasion of MM by suppressing CADM1 expression. Our findings elucidate a new mechanism for LNMAT1-mediated invasion-metastasis cascade in MM and suggest that LNMAT1 may be a new therapeutic target and prognostic predictor for MM.

## Introduction

It is estimated that there will be 96,480 newly diagnosed malignant melanoma (MM) cases in 2019 in the United States following a drastically increased incidence throughout the last decade (1). Despite continuous improvement in diagnosis and treatment, the current recommended maintenance schedules, from radical resection to molecular-targeted drugs, are only effective in a subset of patients (2, 3). Tumors spread to distant sites or visceral organs in some early diagnosed patients, and the 5-year overall survival rate remains extremely disappointing for this subset of patients (4). Hence, it is highly important to explore new detailed mechanisms that account for the invasion-metastasis cascade in MM.

Long non-coding RNAs (lncRNAs) are reported to play pivotal roles in a wide range of vital biological processes (5, 6). More importantly, lncRNAs have also been identified as crucial regulators in the metastasis of multiple cancer types and function as oncogenes or tumor suppressors depending on the cancer type or circumstance (7). Regarding MM, it was reported that lncRNAs could function as molecular scaffolds to regulate the expression levels and functions of established oncogenes or tumor suppressors by interacting with RNA-binding proteins (8–10). Nevertheless, only a few lncRNAs have been functionally characterized in MM, and the mechanisms underlying their biological functions are yet to be fully elucidated. Long intergenic non-coding RNA 01296 (Linc01296), also known as lymph node metastasis associated transcript 1 (LNMAT1), is located in chromosome 14q11.2 and was identified as a metastasis-promoting gene in multiple cancer types (11, 12). It was reported that LNMAT1 could promote invasion and metastasis by either functioning as a ceRNA (13) or by interacting with RNA-binding proteins (14). However, the expression pattern and functions of LNMAT1 in MM remain unclear.

CADM1, a member of the cell adhesion molecule family, has been proven to be a tumor suppressor in many cancers, including breast cancer (15), esophageal squamous cell carcinoma (16), and hepatocellular carcinoma (17). In MM, it was reported that the expression of CADM1 was also significantly downregulated and functions as a tumor suppressor by suppressing matrix metalloproteinases (MMPs) in MM (18, 19). However, the regulating mechanism of CADM1 in MM is not fully elucidated.

In the current study, we determined that LNMAT1 was upregulated in MM tissues and cells, with enhanced expression in patients with lymph node metastasis and metastatic MM cell lines. More importantly, we found that LNMAT1 inhibited invasion and lung metastasis by suppressing CADM1 expression by recruiting EZH2 to its promoter. Our study indicates that LNMAT1 promotes the invasion-metastasis cascade and may be a potential therapeutic target in MM.

## Materials and Methods

### Clinical Specimens

This study was carried out in accordance with the recommendations of Ethical Committee of the First Affiliated Hospital of Xi'an Jiaotong University. The protocol was approved by the Ethical Committee of the First Affiliated Hospital of Xi'an Jiaotong University. All subjects gave written informed consent in accordance with the Declaration of Helsinki. A total of 13 human MM tissues of diagnosed MM patients and 13 benign nevi (BN) tissues of healthy controls were resected and collected at the First Affiliated Hospital of Xi'an Jiaotong University from 2010 to 2017. Written informed consent was obtained from the participants enrolled in this study. Detailed information about the MM patients is provided in Supplementary Table 1.

### Cell Culture

Human malignant melanoma cell lines WM35, A375, A2058, and mouse malignant melanoma cell line B16/F10 were purchased from GeneChem (Shanghai, China), and human epidermal melanocytes HEMa-LP were purchased from ThermoFisher (ThermoFisher, MA, USA). HEMa-LP cells were cultured in Medium 254 (ThermoFisher, MA, USA) supplemented with human melanocyte growth supplement, and WM35, A375, A2058, and B16/F10 cells were cultured in Dulbecco's modified eagle medium (DMEM, Gibco, NY, USA) supplemented with 10% fetal bovine serum (Cellmax, Beijing, China) at 37°C in a humidified atmosphere of 5% CO_2_.

### shRNA Infection and siRNA Transfection

Lentiviral small hairpin RNA (shRNA) directed against LNMAT1 in human and mouse-derived cells and scrambled negative control (NC) shRNA were designed and provided by GeneChem (Shanghai, China). Briefly, the LNMAT1 shRNAs targeting human LNMAT1 and NC shRNAs were cloned into the Bam I and Age I sites of the CV146 core vector (Ubi-MCS-SV40-firefly-Luciferase-IRES-Puromycin). The LNMAT1 shRNAs targeting mouse LNMAT1 and NC shRNAs were cloned into the Bam I and Age I sites of the GV260 core vector (hU6-MCS-Ubi-firefly-Luciferase-IRES-Puromycin). Then, 20 μg CV146-LNMAT1 shRNAs/NC shRNAs for human LNMAT1 knockdown or GV246-LNMAT1 shRNA/NC shRNAs for mouse LNMAT1 knockdown along with lentiviral packaging helper plasmid Helper 1.0 (15 μg) and Helper 2.0 (10 μg) were co-transfected into 293T cells by Lipofectamine 2000. The cell supernatant was collected 48 h later and then centrifuged to concentrate and purify human and mouse LNMAT1 shRNAs and NC shRNAs. LNMAT1 siRNA, CADM1 siRNA, and scrambled NC siRNA were synthesized and provided by Ribobio (Guangzhou, China). The sequences for shRNAs and siRNAs used in our study are provided in Supplementary Table 2.

For shRNA infection, MM cells were seeded in six-well plates and infected by HiTransG A (Genechem) according to the manufacturer's protocol. Then, MM cells were selected with puromycin for 2 weeks to remove uninfected MM cells and obtain stable LNMAT1 knockdown cells. The stable LNMAT1 knockdown MM cells were collected for qRT-PCR, western blot (WB), transwell assays, wound healing assays, and animal experiments.

For siRNA transfection, MM cells were seeded in six-well plates and transfected by Lipofectamine 2000 (ThermoFisher) according to the manufacturer's protocol. After 48 h, cells were collected for qRT-PCR, WB, transwell, and wound healing assays.

### Migration and Invasion Assays, Wound Healing Assays, and WB

The number of migratory and invasive cells with LNMAT1 NC and LNMAT1 shRNA were measured by transwell assay (Corning, NY, USA) with or without Matrigel (BD, CA, USA), and the migratory distance of cells with LNMAT1 NC and LNMAT1 shRNA was measured by wound healing assays. CADM1 protein levels in MM cells infected with LNMAT1 NC and LNMAT1 shRNA were measured by WB. All procedures for transwell assays, wound healing assays, and WB were performed as described in our previous study (20).

### Chromatin Immunoprecipitation (ChIP)-qPCR Assay

ChIP-qPCR assays were performed using the EZ-Magna ChIP A/G kit (Millipore, MA, USA) following the manufacturer's instructions. First, 1 × 10^6^ MM cells were fixed in formaldehyde for 10 min, cell lysates were sonicated and sheared to generate chromatin DNA between 100 and 200 bp in length, and then the lysates were immunoprecipitated with anti-EZH2 (Cell Signaling Technology, USA) or anti-H3K27me3 (Abcam, UK). IgG served as the control. Then, the precipitated chromatin DNA was analyzed by qRT-PCR.

### RNA Immunoprecipitation (RIP)

RIP assays were performed using the EZ-Magna RIP kit (Merck Millipore, USA) following the manufacturer's protocol. MM cells were lysed in RIP lysis buffer, and cell extracts were incubated with anti-EZH2 (Cell Signaling Technology, USA) or IgG for 6 h. Then, purified RNA was analyzed by qRT-PCR to identify the presence of LNMAT1.

### Quantitative Real-Time Polymerase Chain Reaction (qRT-PCR)

RNA extraction and qRT-PCR were performed as described in our previous study. The primers used in this study were purchased from Sangon Biotech (Shanghai, China) and are displayed in Supplementary Table 3.

### Animal Experiment

This study was carried out in accordance with the recommendations of Animal Care and Use Committee of Xi'an Jiaotong University. For lung metastasis assays, B16/F10 cells were seeded in six-well plates, and LNMAT1 shRNA targeting mouse LNMAT1 was infected by HiTransG A (Genechem) according to the manufacturer's protocol. Then, B16/F10 cells were selected with puromycin for 2 weeks to remove uninfected B16/F10 cells and obtain stable LNMAT1 knockdown B16/F10 cells. LNMAT1 stably silencing B16/F10 cells (1 × 10^6^) or control cells were injected into the tail vein of 6-week-old C57/B6 mice (Animal Center of Xi'an Jiaotong University, Xi'an, China; *n* = 5 for each group). All mice were housed and maintained under specific pathogen-free conditions, and all experiments were approved by the Animal Care and Use Committee of Xi'an Jiaotong University and performed in accordance with institutional guidelines. Lung metastases were monitored and quantified by the Xenogen IVIS Kinetic Imaging System (PerkinElmer, MA, USA).

### Statistical Analysis

IBM SPSS statistical software (version 22.0) was used to perform statistical analyses. Student's *t*-test was used for data analysis, and *P*-values were determined using 2-sided tests. *P* < 0.05 was considered to have statistical significance.

## Results

### LNMAT1 Is Upregulated in MM Cells and Tissues With High Metastasis Potential

**Figure 1:**
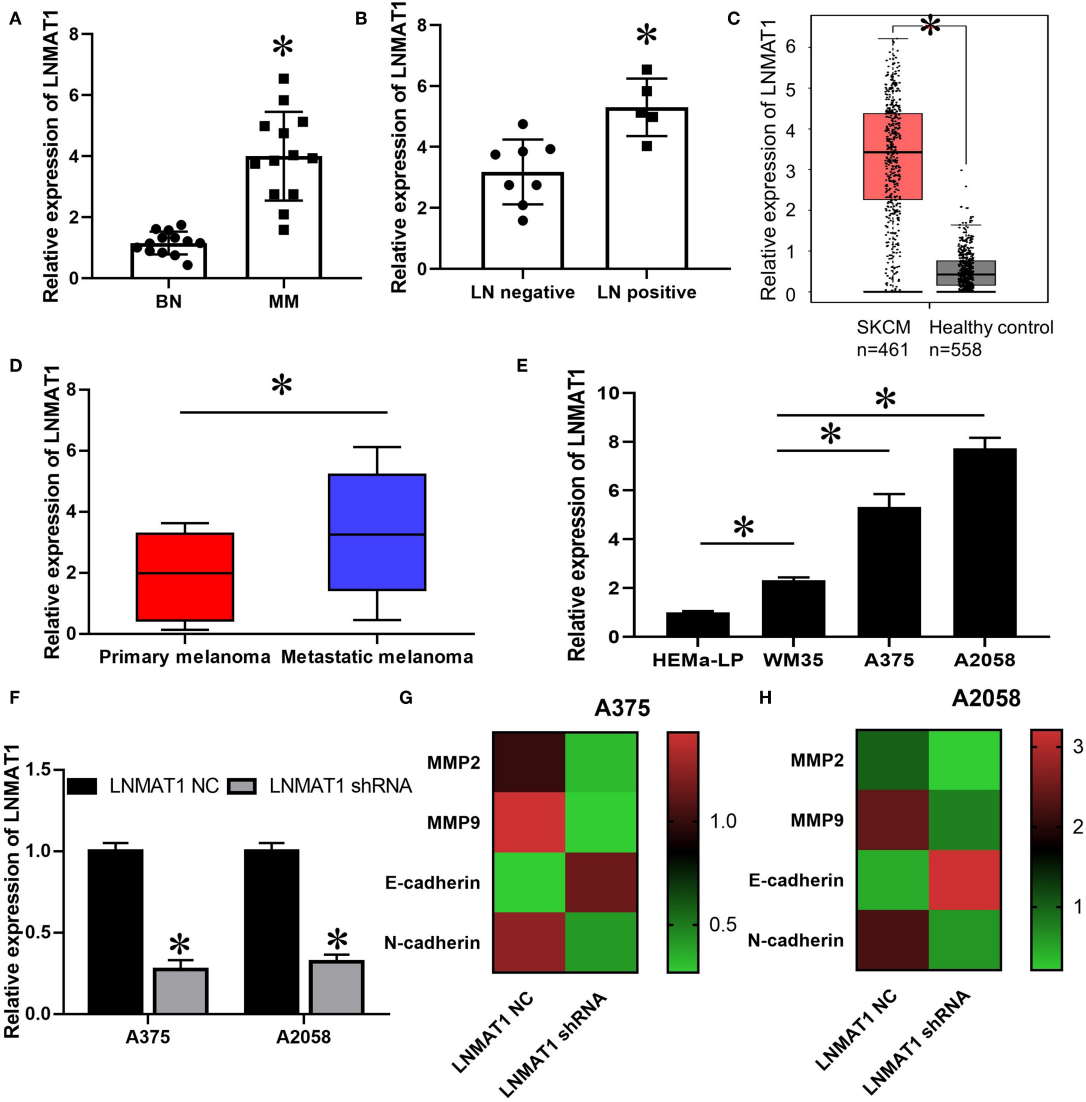
LNMAT1 is upregulated in MM cell and tissues with high metastasis potential. **(A)** The expression of LNMAT1 in BN and MM (8 cutaneous melanoma and 5 acral melanoma); **(B)** The expression of LNMAT1 in MM patients with or without lymph node metastasis; **(C)** The expression of LNMAT1 in 461 patients with skin cutaneous malignant melanoma (SCKM, red box) and 558 healthy controls (Benign nevi (*n* = 346) and normal skin (*n* = 212), black box). TCGA data was analyzed by GEPIA (http://gepia.cancer-pku.cn/). **(D)** The expression of LNMAT1 in 101 patients with primary cutaneous MM (Red box) and 360 patients with metastatic cutaneous MM (Blue box). TCGA data was analyzed by GEPIA (http://gepia.cancer-pku.cn/). **(E)** The expression of LNMAT1 in human epidermal melanocytes and primary and metastatic MM cells; **(F)** The silencing effects of LNMAT1 shRNA in MM cells; **(G,H)** The expression of MMP-2, MMP-9, E-cadherin, and N-cadherin in A375 **(G)** and A2058 **(H)** cells infected with LNMAT1 shRNA or NC. Data are shown as the mean ± S.E. *P < 0.05, ns: not significant. The representative results of three independent experiments are shown.

qRT-PCR analysis indicated that LNMAT1 is upregulated in MM tissues (cutaneous and acral melanoma) compared to BN tissues (Figure 1A, *P* < 0.05). Furthermore, LNMAT1 was found in higher levels in MM patients with lymph node (LN) metastasis than those without LN metastasis (Figure 1B, *P* < 0.05). Accordingly, the TCGA database from GEPIA (http://gepia.cancer-pku.cn) also showed enhanced LNMAT1 expression in MM (*P* < 0.05, Figure 1C), and enhanced LNMAT1 levels were observed in metastatic MM (*P* < 0.05, Figure 1D). Furthermore, LNMAT1 was also higher in MM cells than in HEMa-LP cells, and enhanced LNMAT1 levels were observed in MM cell lines with high metastatic potential (A375 and A2058) compared to primary MM cells (Figure 1E, *P* < 0.05). More importantly, after silencing LNMAT1 expression in MM cells with shRNAs (Figure 1F, *P* < 0.05), the mRNA expression levels of MMP-2, MMP-9, and N-cadherin, which are markers of tumor invasion-metastasis cascade, were found to be downregulated by qRT-PCR. Meanwhile, the expression of E-cadherin, one of the most important tumor metastasis suppressors and epithelial–mesenchymal transition (EMT) markers, was upregulated (Figures 1G,H). These results indicated that LNMAT1 may play an oncogenic role in the invasion-metastasis cascade in MM.

### LNMAT1 Promotes the Invasion-Metastasis Cascade in MM *in vitro* and *in vivo*

**Figure 2:**
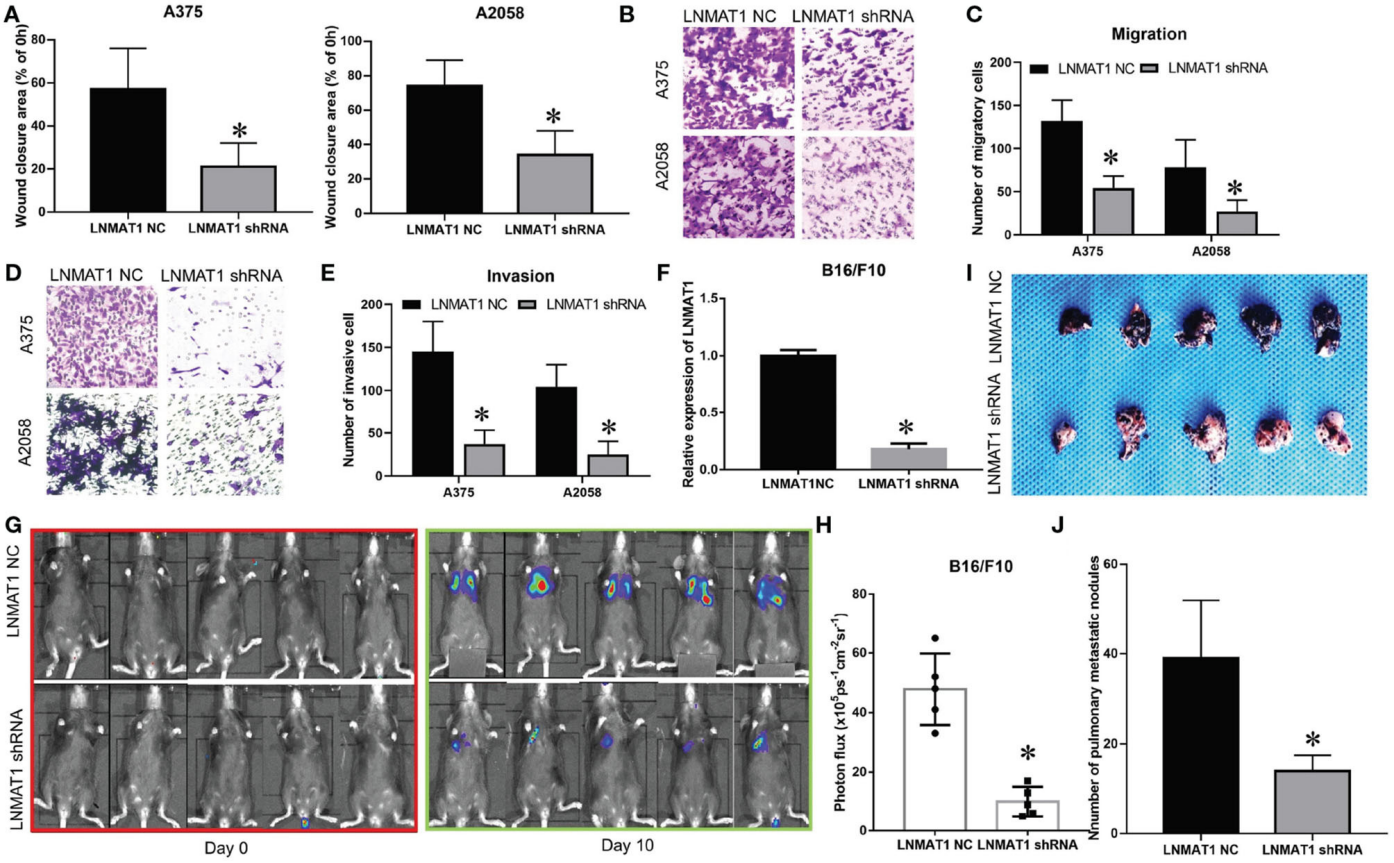
LNMAT1 promotes the invasion-metastasis cascade in MM *in vitro* and *in vivo*. **(A)** MM cells were infected with LNMAT1 NC or shRNA. Wound healing assays were performed to detect the migratory distance in the indicated groups. The representative results of three independent experiments are shown; **(B,C)** Transwell assays were performed to detect the number of migratory cells in the indicated groups. The representative results of three independent experiments are shown; **(D,E)** Transwell assays were performed to detect the number of invasive cells in the indicated groups. The representative results of three independent experiments are shown; **(F)** The silencing effects of LNMAT1 shRNA in B16/F10 cells; **(G,H)** Bioluminescence images at day 0 and day 10 **(G)** and statistical analysis **(H)** of bioluminescence at day 10 (lung colonization) in C57/B6 mice (*n* = 5) that were intravenously injected with LNMAT1 stably silencing B16/F10 cells or NC cells; **(I,J)** Bright field imaging **(I)** and number of metastatic nodules **(J)** at day 10 in the lungs of C57/B6 mice (*n* = 5) that were intravenously injected with LNMAT1 stably silencing B16/F10 cells or NC cells. Data are shown as the mean ± S.E. *P < 0.05, ns: not significant.

To further elucidate the functions of LNMAT1 in the invasion-metastasis cascade of MM, wound healing, transwell, and B16/F10 pulmonary metastasis models were employed. Wound healing assays demonstrated that the migratory distance was decreased in MM cells infected with LNMAT1 shRNA lentivirus compared to that in control cells (Figure 2A, *P* < 0.05). Furthermore, silencing LNMAT1 expression in MM cells could attenuate cell migratory and invasive abilities (Migration assays: Figures 2B,C, invasion: Figures 2D,E; all *P* < 0.05), Then, we infected B16/F10 cells with LNMAT1 shRNA to stably silence LNMAT1 expression in B16/F10 and investigate the effects of LNMAT1 on lung metastasis in B16/F10 *in vivo* (Figure 2F). As shown by the Bioluminescence imaging (BLI) data (Figures 2G,H) and *ex vivo* photography of lung tissues (Figures 2I,J), LNMAT1 depletion significantly decreased lung colonization of B16/F10; this was consistent with the *in vitro* results. These results indicated that LNMAT1 promotes the migration and invasion of MM *in vitro* and *in vivo*.

### CADM1 Is the Downstream Target of LNMAT1 in MM

**Figure 3:**
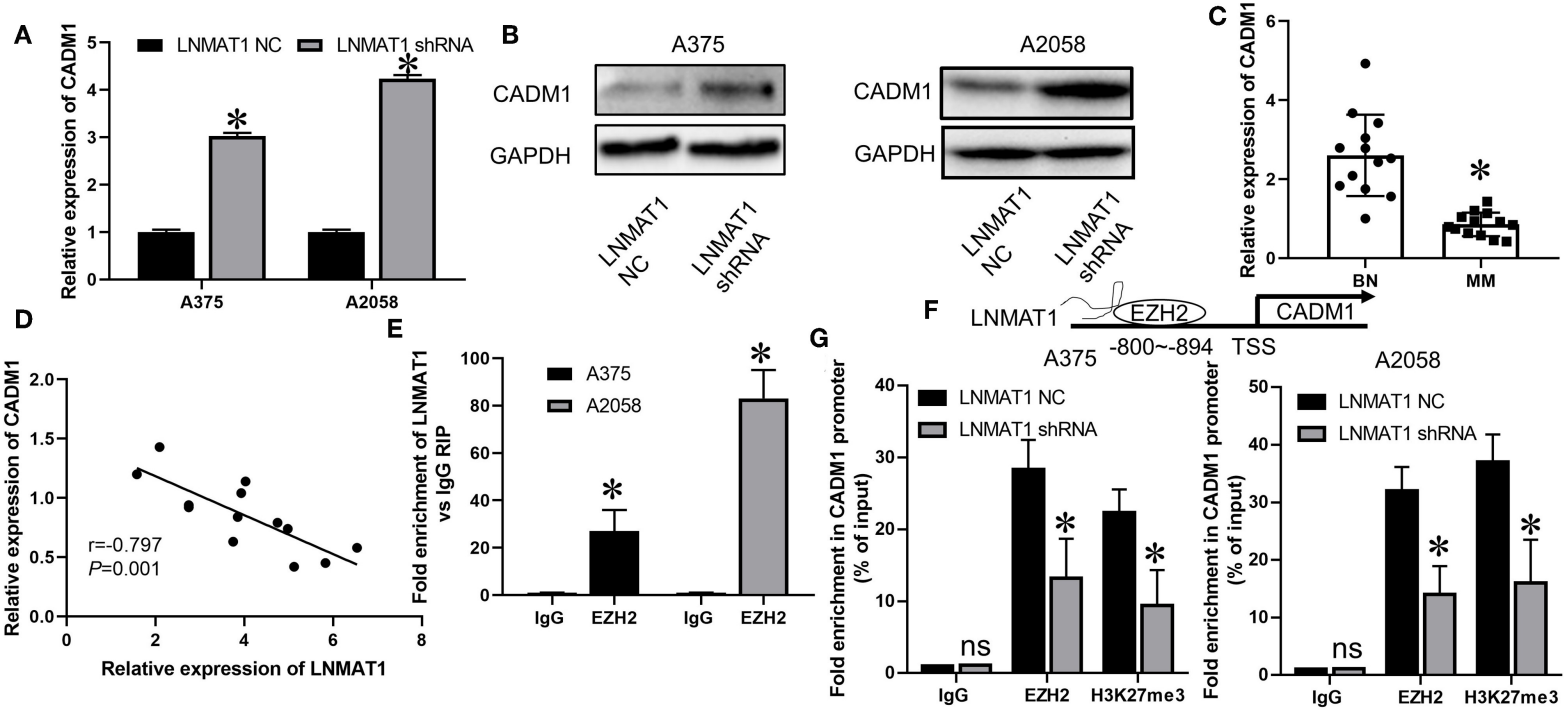
LNMAT1 epigenetically suppresses CADM1 expression by recruiting EZH2 to its promoter. **(A,B)** The mRNA **(A)** and protein **(B)** levels of CADM1 in response to LNMAT1 silencing were detected in MM cells by qRT-PCR and WB; **(C)** The relative expression of CADM1 was detected in BN and MM tissues of by qRT-PCR; **(D)** Correlations between CADM1 and LNMAT1 expression in MM tissues; **(E)** RIP with anti-EZH2 and IgG from extracts of MM cells infected with LNMAT1 NC or shRNA and qRT-PCR were performed to confirm the interactions between LNMAT1 and EZH2/IgG; **(F)** Schematic diagram showing mechanism of LNMAT1 at the EZH2 binding site on LNMAT1 gene promoters. **(G)** ChiP-qPCR assays of MM cells infected with LNMAT1 NC or shRNA were performed to reveal the effects of LNMAT1 on EZH2 and H3K27me3 occupancy in the CADM1 promoter. Data are shown as the mean ± S.E. *P < 0.05, ns: not significant. The representative results of three independent experiments are shown.

Previously, it was found that CADM1, an established metastasis suppressor gene, could inhibit cell migration and invasion in MM by suppressing the expression of MMP-2 and MMP-9 (18). Thus, we investigated whether a regulatory mechanism existed between LNMAT1 and CADM1 in MM. As shown by qRT-PCR and WB, mRNA (Figure 3A, *P* < 0.05) and protein (Figure 3B) levels of CADM1 were enhanced after silencing LNMAT1 expression in MM cells. Additionally, CADM1 expression was downregulated in MM tissues compared to BN tissues (Figure 3C, *P* < 0.05). More importantly, CADM1 expression was inversely correlated with LNMAT1 expression in MM tissues (Figure 3D, *P* < 0.05).

### LNMAT1 Epigenetically Suppresses CADM1 Expression by Recruiting EZH2 to Its Promoter

It has been reported that LNMAT1 could epigenetically suppress KLF2 expression by interacting with EZH2, an RNA-binding protein and crucial regulator for the trimethylation of histone H3 at lysine 27 (H3K27me3) (14). Thus, we hypothesized that LNMAT1 might suppress CADM1 expression by recruiting EZH2 to its promoter. RIP assays determined that LNMAT1 could directly bind with EZH2 in MM cells (Figure 3E, *P* < 0.05). Chip-qPCR assays further revealed that LNMAT1 depletion decreased EZH2 binding and H3K27me3 modification in the CADM1 promoter (Figures 3F,G; *P* < 0.05). These results indicated that LNMAT1 suppressed CADM1 expression by recruiting EZH2 to its promoter and inducing the modification of histone methylation to mediate epigenetic silencing.

### CADM1 Mediates the Function of LNMAT1 in MM Cells

**Figure 4:**
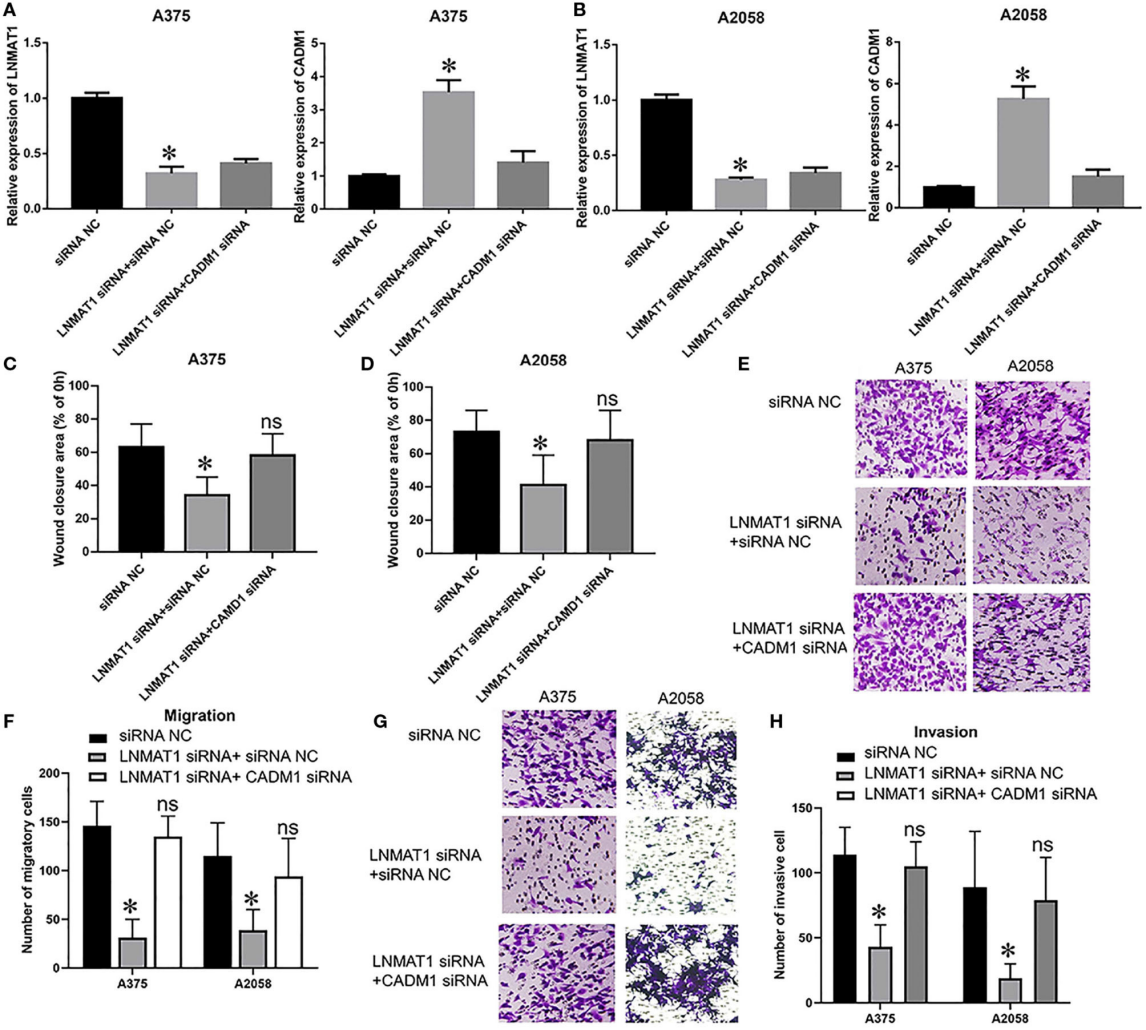
CADM1 mediates the function of LNMAT1 in MM cells. MM cells were transfected with siRNA NC, LNMAT1 siRNA + siRNA NC, or LNMAT1 siRNA + CADM1 siRNA. **(A,B)** LNMAT1 and CADM1 mRNA expression levels in A375 **(A)** and A2058 **(B)** cells of the indicated groups; **(C,D)**. Wound healing assays were used to determine the migratory distances of A375 **(C)** and A2058 **(D)** cells in the indicated groups; **(E,F)** Transwell assays were used to determine the number of migratory A375 **(E)** and A2058 **(F)** cells in the indicated groups; **(G,H)** Transwell assays were used to determine the number of invasive A375 **(G)** and A2058 **(H)** cells in the indicated groups. Data are shown as the mean ± S.E. *P < 0.05, ns: not significant. The representative results of three independent experiments are shown.

Lastly, we performed rescue experiments to further identify whether LNMAT1 promoted the invasion-metastasis cascade by inhibiting CADM1 expression. Overall, qRT-PCR (Figures 4A,B; all *P* < 0.05), wound healing (Figures 4C,D; all *P* < 0.05), and transwell assays (Migration: Figures 4E,F, invasion: Figures 4G,H; all *P* < 0.05) demonstrated that silencing CADM1 expression could partly rescue the inhibitory effects on cell migration and invasion induced by LNMAT1 depletion in MM cells. These results indicated that CADM1 mediates the function of LNMAT1 in MM.

## Discussion

LncRNAs have been identified as crucial regulators and biomarkers in multiple cancers, including MM, and LNMAT1 has been confirmed as an oncogenic lncRNA in various cancers. In non-small-cell lung cancer and cholangiocarcinoma, LNMAT1 could function as a competitive endogenous RNA (ceRNA) and promote cell proliferation and migration by sponging miR-5095 (21, 22). In colorectal cancer, LNMAT1 promoted liver metastasis and tumorigenesis and activated the PI3K/AKT cascade by competitively binding with miR-26a (23). In esophageal squamous cell carcinoma, LNMAT1 promoted cell proliferation and invasion by epigenetically suppressing KLF2 (14). In bladder cancer, LNMAT1 was found to be upregulated in patients with lymph node metastasis and was a potential lymphatic metastasis promoter. Mechanistic experiments confirmed that LNMAT1 upregulated CCL2 expression by interacting with hnRNPL and enhancing H3K4me3 modification (11). Consistent with these previous studies, we also identified that LNMAT1 plays a role in promoting metastasis in MM. We found that LNMAT1 was upregulated in MM tissues and cells compared to those of BN and melanocytes. Moreover, we also found that LNMAT1 expression was further upregulated in patients with lymph node metastasis and cells with highly metastatic potential. Lastly, we determined that silencing LNMAT1 inhibited cell migration and invasion in MM *in vitro* and *in vivo*. Combined with previous studies concerning the functions of LNMAT1, our study further confirmed that LNMAT1 plays an oncogenic role in carcinogenesis and cancer progression.

CADM1 expression is relatively lower in metastatic breast cancer (24) and lung adenocarcinoma (25) patients than in patients with non-invasive cancer. In MM, it was reported that CADM1 expression was significantly downregulated in melanoma tissues (19). Furthermore, CADM1 upregulation inhibited MM cell motility and invasiveness (18). It was also found that the expression levels of matrix metalloproteinase-2 (MMP-2) and matrix metalloproteinase-9 (MMP-9) were downregulated by CADM1 over-expression (18). As MMP-2 and MMP-9 are key regulators of extracellular matrix (ECM) degradation and tumor invasion (26), CADM1 is therefore a metastasis susceptibility gene and is involved in the invasion-metastasis cascade in MM. In the current study, we found that silencing LNMAT1 resulted in the inhibition of MMP-2 and MMP-9 expression. An increase in E-cadherin and decrease in N-cadherin were also found after silencing LNMAT1 in MM, indicating that LNMAT1 potentially plays an important role in EMT and the invasion-metastasis cascade in MM. As both LNMAT1 and CADM1 are key regulators of MMP expression and EMT and induced the invasion-metastasis cascade, we further investigated the relationships between CADM1 and LNMAT1 in MM. We found that LNMAT1 expression was inversely correlated with CADM1 in MM tissues and cells. In addition, LNMAT1 could suppress CADM1 expression by recruiting EZH2 to its promoter and induce modifications of histone methylation. Thus, we conclude that the metastasis-promoting role of LNMAT1 in MM is mediated by CADM1 suppression.

In conclusion, we revealed that LNAMT1 is a potential oncogene in MM. LNAMT1 was upregulated in MM tissues and cells with highly metastatic potential. Furthermore, LNAMT1 could promote the invasion-metastasis cascade of MM *in vivo* and *in vitro*. Studies on the underlying mechanism showed that LNAMT1 epigenetically suppressed CADM1 expression by recruiting EZH2 to its promoter, and silencing CADM1 expression rescued the inhibitory effects on MM cell migration and invasion induced by LNAMT1 depletion. Our study helps to reveal the regulatory mechanism of LNMAT1 and CADM1 in MM and may provide a novel target for MM treatment in the future.

## Data Availability

All datasets generated for this study are included in the manuscript and/or the Supplementary Files.

## Ethics Statement

This study was carried out in accordance with the recommendations of institutional guideline, Ethnical committee of the First Affiliated Hospital of Xi'an Jiaotong University. The protocol was approved by the Ethnical committee of the First Affiliated Hospital of Xi'an Jiaotong University. All subjects gave written informed consent in accordance with the Declaration of Helsinki. The animal experiments were carried out in accordance with the recommendations of institutional guideline, Animal Care and Use Committee of Xi'an Jiaotong University. The protocol was approved by the Animal Care and Use Committee of Xi'an Jiaotong University.

## Author Contributions

KM, XZ, RG, and XM performed the research. LW designed the research study. YZ contributed essential reagents or tools. DH analyzed the data. LW wrote the paper. All authors have read and approved the final manuscript.

### Conflict of Interest Statement

The authors declare that the research was conducted in the absence of any commercial or financial relationships that could be construed as a potential conflict of interest.
