# Corrigendum: LNMAT1 Promotes Invasion-Metastasis Cascade in Malignant Melanoma by Epigenetically Suppressing CADM1 Expression

**DOI:** 10.3389/fonc.2022.905360

**Published:** 2022-05-10

**Authors:** Kuanhou Mou, Xiang Zhang, Xin Mu, Rui Ge, Dan Han, Yan Zhou, Li-Juan Wang

**Affiliations:** ^1^ Department of Dermatology, First Affiliated Hospital of Xi’an Jiaotong University, Xi’an, China; ^2^ Department of Clinical Laboratory Medicine, Xijing Hospital, Fourth Military Medical University, Xi’an, China

**Keywords:** LNMAT1, EZH2, CADM1, invasion-metastasis cascade, malignant melanoma

Following the publication of the original article, the authors found a few unintentional errors in **Figures 2D, G**. The authors clarify that corrections were made in **Figures 2D, G** panels, as follows: (1) an image overlap was corrected in **Figure 2D**, more specifically in the representative images of invasive A375 cells between LNMAT1 shRNA (**Figure 2D**) when compared to LNMAT1 siRNA + siRNA NC (**Figure 4G**). The representative image of invasive A375 cells of LNMAT1 siRNA group + siRNA NC shown in **Figure 4G** is accurate, and the representative image of invasive A375 cells of LNMAT1 shRNA shown in **Figure 2D** is now corrected; (2) In **Figure 2G**, the images of Day 0 and Day 10 were corrected, since there were overlapping issues between different images. The corrected figures appear below.

**Figure 2:**
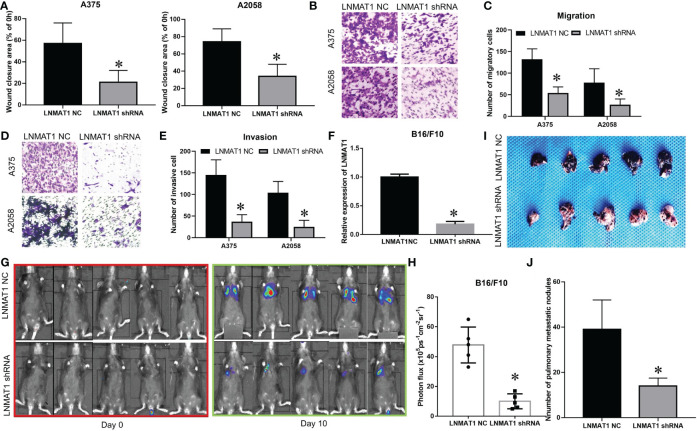
LNMAT1 promotes the invasion-metastasis cascade in MM in vitro and in vivo. **(A)** MM cells were infected with LNMAT1 NC or shRNA. Wound healing assays were performed to detect the migratory distance in the indicated groups. The representative results of three independent experiments are shown; **(B, C)** Transwell assays were performed to detect the number of migratory cells in the indicated groups. The representative results of three independent experiments are shown; **(D, E)** Transwell assays were performed to detect the number of invasive cells in the indicated groups. The representative results of three independent experiments are shown; **(F)** The silencing effects of LNMAT1 shRNA in B16/F10 cells; **(G, H)** Bioluminescence images at day 0 and day 10 **(G)** and statistical analysis **(H)** of bioluminescence at day 10 (lung colonization) in C57/B6 mice (n = 5) that were intravenously injected with LNMAT1 stably silencing B16/F10 cells or NC cells; **(I, J)** Bright field imaging **(I)** and number of metastatic nodules **(J)** at day 10 in the lungs of C57/B6 mice **(n = 5)** that were intravenously injected with LNMAT1 stably silencing B16/F10 cells or NC cells. Data are shown as the mean ± S.E. *P < 0.05, ns, not significant.

**Figure 4:**
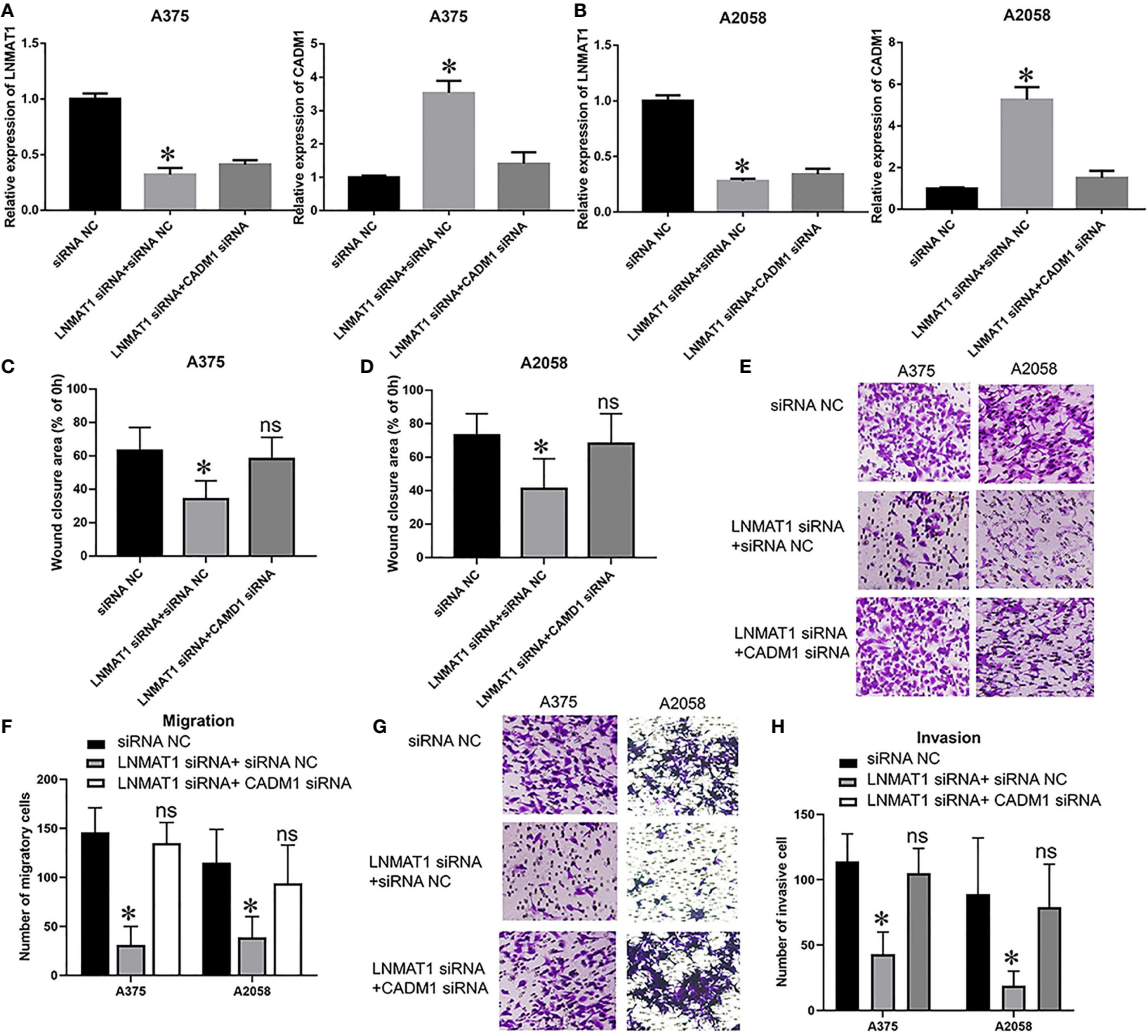
CADM1 mediates the function of LNMAT1 in MM cells. MM cells were transfected with siRNA NC, LNMAT1 siRNA + siRNA NC, or LNMAT1 siRNA + CADM1 siRNA. **(A, B)** LNMAT1 and CADM1 mRNA expression levels in A375 **(A)** and A2058 **(B)** cells of the indicated groups; **(C, D)**. Wound healing assays were used to determine the migratory distances of A375 **(C)** and A2058 **(D)** cells in the indicated groups; **(E, F)** Transwell assays were used to determine the number of migratory A375 **(E)** and A2058 **(F)** cells in the indicated groups; **(G, H)** Transwell assays were used to determine the number of invasive A375 **(G)** and A2058 **(H)** cells in the indicated groups. Data are shown as the mean ± S.E. *P < 0.05, ns, not significant. The representative results of three independent experiments are shown.

The authors apologize for this error and state that this does not change the scientific conclusions of the article in any way. The original article has been updated.

## Publisher’s Note

All claims expressed in this article are solely those of the authors and do not necessarily represent those of their affiliated organizations, or those of the publisher, the editors and the reviewers. Any product that may be evaluated in this article, or claim that may be made by its manufacturer, is not guaranteed or endorsed by the publisher.

