# Supplementary material for: LNMAT1 Promotes Invasion-Metastasis Cascade in Malignant Melanoma by Epigenetically Suppressing CADM1 Expression
Source: Front Oncol. 2019 Jul 3;9:569. doi: 10.3389/fonc.2019.00569 (PMC6617740; doi:10.3389/fonc.2019.00569)
Supplement: Supplementary file 1 [file Table_1.DOCX]

**Supplementary table 1. Clinicopathological features of MM patients in this study**

| Number | Age | Gender | Clinical stage | Anatomic site | Lymph nodes metastasis | Distant metastasis |
| --- | --- | --- | --- | --- | --- | --- |
| 1 | 46 | Female | IV | Acral | Positive | Positive |
| 2 | 52 | Male | II | Trunk | Negative | Negative |
| 3 | 45 | Female | III | Other | Positive | Negative |
| 4 | 66 | Male | III | Trunk | Positive | Negative |
| 5 | 61 | Female | II | Acral | Negative | Negative |
| 6 | 65 | Female | III | Trunk | Positive | Negative |
| 7 | 54 | Female | II | Acral | Negative | Negative |
| 8 | 41 | Male | I | Other | Negative | Negative |
| 9 | 51 | Male | IV | Trunk | Positive | Positive |
| 10 | 63 | Female | II | Acral | Negative | Negative |
| 11 | 42 | Male | II | Trunk | Negative | Negative |
| 12 | 61 | Male | IV | Other | Negative | Positive |
| 13 | 80 | Male | II | Acral | Negative | Negative |

**Supplementary table 2. shRNAs and siRNAs used in the experiments**

| Name | Sequence |
| --- | --- |
| LNMAT1 shRNA (human) sense | 5’-GATCCTATGATACATTTGTGTTAACTCGAGTTAACACA-AATGTACATATGTTTTTG-3’ |
| LNMAT1 shRNA (human) antisense | 5’-AATTCAAAAACATATGTACATTTGTGTTAACTCG-AGTTAACACAAATGTGCATGTG-3’ |
| LNMAT1 shRNA (mouse) sense | 5’-GATCCTATGATGTGCAGAGTTTATTAACTCTTAACACA-AATGTACATATGTTTTTG-3’ |
| LNMAT1 shRNA (mouse) antisense | 5’-AATTCAAAAATTCAGTATATGTGTCATTAACTCG-AGTTAACACAAATGTGCATGTG-3’ |
| NC shRNA (human) sense | 5’-GATCCTATAGACAATCCAACCAACCAGTTAACTGGT-GTGGTTGGATTGTCTATTTTTG-3; |
| NC shRNA (human) antisense | 5’-AATTCAAAAATAGACAATCCAACCAACCACAGTTAAC-  TGGTTGGTTGGATTGTCTAG-3’ |
| NC shRNA (mouse) sense | 5’-GATCCTCATAGACAAAATCCACACCAGTTAACTGGT-GTGGTTGGATTGTCTATTTTTG-3; |
| NC shRNA (mouse) antisense | 5’-AATTCACAATAAATAGGCCAACCTACCTTAACTGGT-GTGGTTGGTTGGATTGTCTAG-3; |
| LNMAT1 siRNA (human) sense | 5′-GGCUGGAGAAUAUUUCCUATTTT-3′ |
| LNMAT1 siRNA (human) antisense | 5’-AAUAGGAAAUAUUCUCCAGCCTT-3; |
| NC siRNA sense | 5’-UUCUCCGAACGUGUCAGGUTT-3’ |
| NC siRNA antisense | 5’-ACCUGACACGUUCGGAGAATT-3’ |

**Supplementary table 3. Primers used in the experiments**

| Name | Sequence |
| --- | --- |
| LNMAT1 forward (human) | 5’-GAGAAGCAGTGGTGGGTTCC-3’ |
| LNMAT1 reverse (mouse) | 5’-GAGCAACACAGATGAACCGC-3’ |
| β-actin forward | 5’- CCA AGG CCAACCGCGAGAAGATGAC-3’ |
| β-actin reverse | 5’-AGGGTACATGGTGGTGCC GCCAGAC -3’ |
| CADM1 forward | 5’-GCAGGTGAAGAAGGCTCGAT-3’ |
| CADM1 reverse | 5’- CCCAGAATGATGAGCAAGCA-3’ |
| E-cadherin forward | 5’-AATAGTGCCTAAAGTGCTGC-3’ |
| E-cadherin reverse | 5’-AGACCCACCTCAATCATCCT-3’ |
| N-cadherin forward | 5’-ATCCTACTGGACGGTTCG-3’ |
| N-cadherin reverse | 5’-TTGGCTAATGGCACTTGA-3’ |
| MMP 2 forward | 5’-CTTCTTCCCTCGCAAGCC-3’ |
| MMP 2 reverse | 5’-ATGGATTCGAGAAAACCG-3’ |
| MMP 9 forward | 5’-ACGCAGACATCGTCATCC-3’ |
| MMP 9 reverse | 5’-AACCGAGTTGAACCACG-3’ |
| LNMAT1 forward (mouse) | 5’-TAATTATGCCTCACAACCATC-3’ |
| LNMAT1 reverse (mouse) | 5’-TCAAGCAGAGGCTGAGAAGT-3’ |
| CADM1-promoter forward | 5’-AGAGACAGGGGAAGCTCG-3’ |
| CADM1-promoter reverse | 5’-TTAGTCAAGGCTTCAGGTGAAGAGC-3’ |
